# A good-practice guide to solving and refining mol­ecular organic crystal structures from laboratory powder X-ray diffraction data

**DOI:** 10.1107/S2053229625008046

**Published:** 2025-09-17

**Authors:** Elena Kabova, Margarita Mersiyanova, Kenneth Shankland, Norman Shankland, Mark Spillman

**Affiliations:** a School of Pharmacy, University of Reading, Reading, United Kingdom; b Independent Researcher, United Kingdom; c CuspAI, Cambridge, United Kingdom; University of Strathclyde, United Kingdom

**Keywords:** mol­ecular crystal structures, crystal structure solution and refinement, powder X-ray diffraction, good practice, *DASH*, *TOPAS-Academic*

## Abstract

This article focuses on a specific real-space methodology for solving and refining mol­ecular organic crystal structures, developed by the authors and collaborators. It outlines a practical route from polycrystalline samples to refined crystal structures, emphasizing efficient global optimization by *DASH* and the robust refinement capabilities of *TOPAS*. The approach prioritizes laboratory-to-laboratory reproducibility *via* a standardized workflow that addresses key challenges in mol­ecular organic crystal structure determination.

## Abstract 2

A step-by-step outline for academic and industrial practitioners determining mol­ecular organic crystal structures from powder X-ray diffraction data.

## Introduction

Powder X-ray diffraction (PXRD) is a foundational technique for characterizing crystalline materials, with patterns serving as fingerprints for phase identification. Crystal structure determination from powder diffraction data (SDPD) originated in the early 20th century, beginning with solutions obtained for various elements using a transmission PXRD set-up with monochromatic incident radiation (Hull, 1917 ▸). The development of the Rietveld refinement method (Rietveld, 1969 ▸) and intensity extraction approaches, including those of Pawley (1981 ▸) and Le Bail (Le Bail *et al.*, 1988 ▸), later provided key com­ponents of pathways to solve structures from PXRD data.

The 1990s marked a turning point for SDPD, as patent disputes over pharmaceutical polymorphs highlighted its value when single crystals were unavailable. However, the low symmetry and large unit cells of active pharmaceutical ingredients (APIs) often result in heavily overlapped PXRD patterns, especially at high 2θ angles. Weak diffraction beyond *ca* 1.5 Å further com­plicates intensity extraction, challenging traditional single-crystal methods. These limitations spurred advances in real-space SDPD techniques (Shankland, 2019 ▸), expanding the range of solvable structures.

The key challenge for SDPD is determining a chemically, crystallographically and energetically sensible structure that fits the observed diffraction data convincingly. Accordingly, accurate SDPD demands rigorous attention to multiple factors, from optimized sample preparation to a verification protocol combining Rietveld refinement and crystal structure geometry optimization, all of which are systematically addressed in this work. The discussion is largely confined to laboratory PXRD, while noting that the majority of steps outlined herein apply equally to data collected, for example, on a high-resolution synchrotron beamline. The software employed by the authors and collaborators in this work is summarized in Table 1 ▸.

## Data collection

### Incident wavelength

Monochromatic Cu *K*α_1_ radiation is recommended for two key reasons:

(i) with scattering intensity proportional to λ^3^, stronger diffraction is obtained with Cu *K*α_1_ (λ = 1.54056 Å) com­pared to Mo *K*α_1_ radiation (λ = 0.70930 Å);

(ii) an incident monochromator eliminates Cu *K*α_2_ and *K*β radiation, ensuring single-peak reflections and avoiding the need for com­putational line stripping.

This monochromation reduces the incident beam intensity relative to a non-monochromated beam, but the resulting longer data collection time is insignificant within the overall SDPD workflow.

All subsequent sections assume the use of monochromatic Cu *K*α_1_ radiation.

### Capillary transmission geometry

The gold standard for SDPD involves collecting data from a sample held in a rotating borosilicate glass capillary, in transmission geometry.^1^ This minimizes the effects of preferred orientation (PO) and ensures optimal beam–sample inter­action for accurate intensity extraction.

The ideal powder particle size (typically 20–50 µm in a 0.7 mm capillary) balances three critical requirements: ensuring homogenous packing, obtaining a true powder average and mitigating PO. A gentle sample grinding step is recommended to achieve an optimal particle size distribution, while avoiding excessive mechanical stress that could induce peak broadening or unintended phase transitions. Overly broad peaks increase reflection overlap, com­plicating indexing (Section 3.2), crystal structure solution (Section 4) and Rietveld refinement (Section 5). Where feasible, recrystallization often yields sharper diffraction peaks, substanti­ally improving the reliability of crystal structure determination.

**Figure 1:**
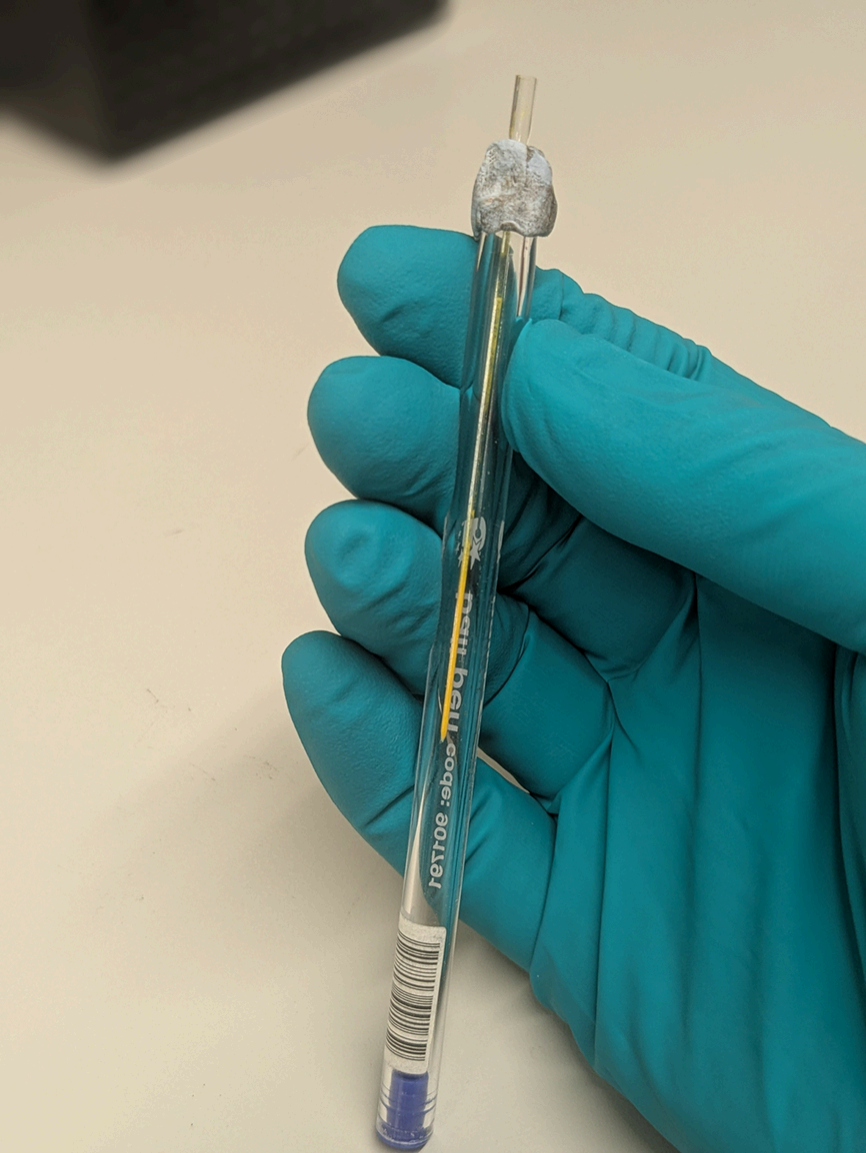
A 0.7 mm borosilicate glass capillary inside the transparent outer casing of a ballpoint pen. The image shows ∼2 cm of packed sample, achieved by scooping a small amount of powder into the bulb of the capillary, then holding the casing upright and tapping it on a hard surface, propelling the powder into the capillary. The most reliable filling is achieved by loading and packing a small amount of powder, multiple times. The fragile capillary is held in place using a ‘Blu Tack’ collar that also cushions it against vigorous tapping. Care must obviously be taken with potent or toxic materials, as the tapping releases small amounts of powder into the atmosphere. When collecting data from a solvate, or a hygroscopic sample, it is prudent to reduce the chance of a phase transition by minimizing capillary void space, sealing the capillary with wax and, optionally, collecting data as a series of short runs. These can then be pooled into a single dataset, provided that there is no evidence of a phase transition.

For instruments where the focal point of the incident beam is on the detector, the capillary diameter does not have a sig­nificant effect upon resolution, *i.e.* the ability of the in­stru­ment to resolve diffraction features. Typically, 0.7 mm diameter (Fig. 1 ▸) is recommended over 0.3 mm (more challenging to fill) and 1.0 mm (requires more sample). If absorption is an issue (rarely the case with mol­ecular organic samples), then a 0.3 mm capillary is recommended.

Modern single-crystal diffractometers equipped with area detectors can also operate in PXRD mode, providing 2D diffraction images that reveal critical sample characteristics. The resulting Debye–Scherrer rings enable direct visualization of PO (manifested as non-uniform intensity distribution around the rings), while also revealing the presence of microcrystals (appearing as discrete Bragg spots superimposed on the rings). This capability offers valuable diagnostic information beyond conventional 1D powder patterns.

### Detectors and alignment

Position-sensitive detectors have long been standard in laboratory PXRD systems, offering superior resolution and count rates com­pared to point detectors. Some also feature energy discrimination, effectively suppressing fluorescence from organometallic samples, most notably those containing Co, Fe or Mn. While peak asymmetry (caused by axial divergence at low 2θ) is well handled by modern software, it is typically minimized during data collection using either a narrow receiving slit or Soller slits placed in front of the detector.

To check alignment, it is good practice to periodically check the instrument zero point by collecting data from a well-characterized sample, over a wide 2θ range, and refining the zero point *via* Pawley fitting. The standard implemented in this work is a sharply-diffracting sample of l-glutamic acid. A well-aligned instrument will have a refined zero point ≤ 0.017° 2θ (where 0.017° 2θ is a typical step size; Section 2.4). A refined zero point > 0.05° 2θ (three step sizes) can be particularly problematic at the powder indexing stage and should be addressed by realigning the instrument.

### Data range and count times

**Figure 2:**
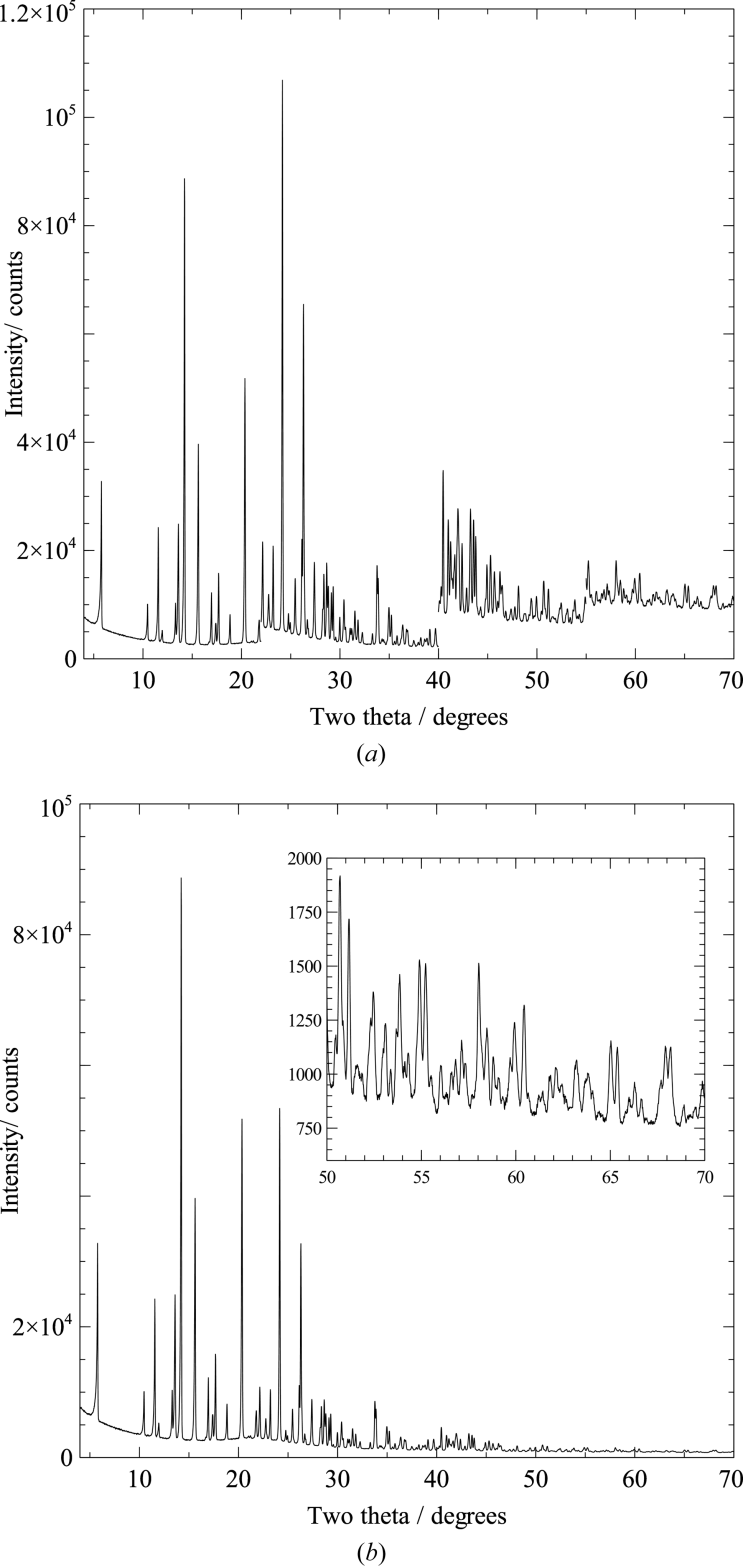
(*a*) Raw VCT data (data ranges as specified in Table 3 ▸) and (*b*) normalized data collected from a sample of morphine sulfate penta­hydrate with a Bruker D8 diffractometer operating in capillary transmission geometry using Cu *K*α_1_ radiation. The inset shows the high quality of the data up to 70° 2θ.

The two recommended data collection schemes for SDPD are shown in Table 2 ▸. The instrumental set-up may not permit data collection at the lower limit of 2θ without a high background from the straight-through beam, but this limit is advantageous for detecting any low-angle reflections corresponding to long cell axes. A two-hour scan, with fixed count time, is generally perfectly adequate for all stages up to and including global optimization. For Rietveld refinement purposes, however, data to higher values of 2θ are required, with at least 1.35 Å real-space resolution desirable. Given the rapid fall-off in diffracted intensity at high values of 2θ, a variable count time (VCT) scheme should be employed to obtain a good signal-to-noise ratio at such high values. While in principle, a continuously increasing count time is ideal, in practice a simple generic scheme (such as that shown in Table 3 ▸) is easier to implement and achieves the desired aim (Fig. 2 ▸). The VCT scheme can be scaled to any desired overall data collection time, which will vary according to irradiated sample volume, sample scattering power, incident beam intensity and the detector used.

### Temperature control and sample degradation

**Figure 3:**
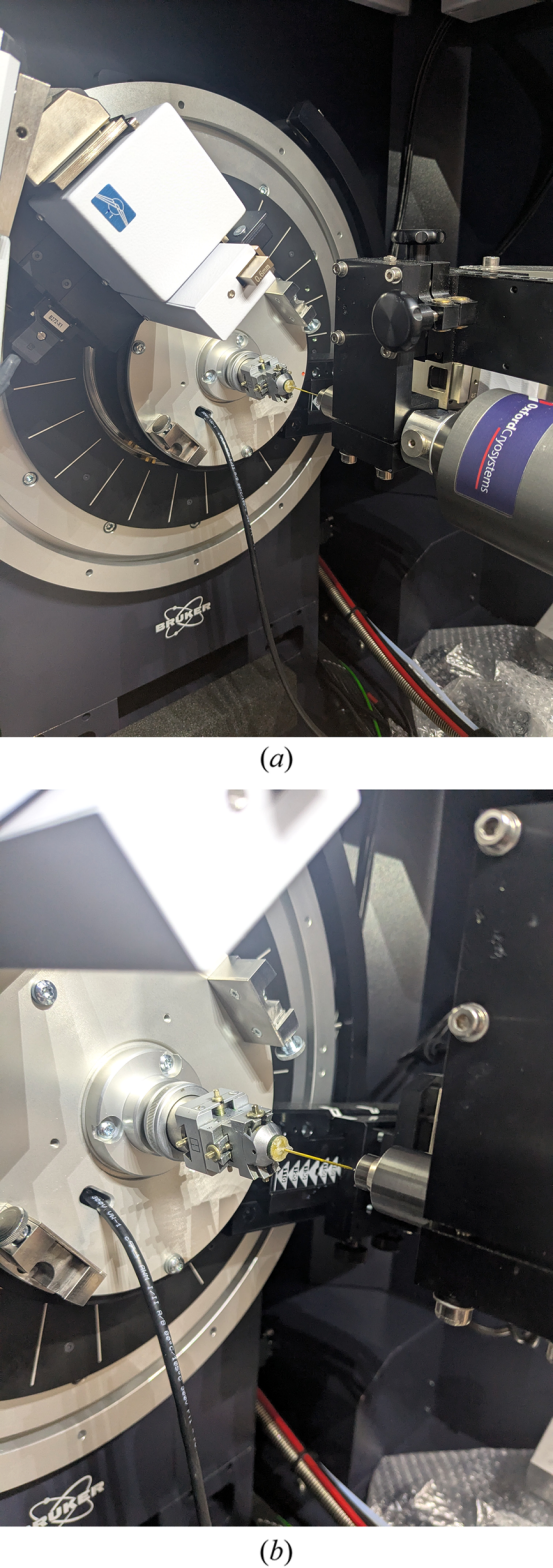
An Oxford Cryosystems Cryostream Compact open-flow sample cooler, mounted coaxially with the rotating sample capillary (*a*) and showing a close-up of the coldhead (*b*).

Low-tem­per­a­ture data collection is not essential, but it is highly advantageous, provided that the sample is not susceptible to a tem­per­a­ture-induced phase transition. Cooling the capillary (∼150 K is recommended) helps mitigate the form-factor fall-off observed in PXRD patterns and significantly improves diffraction data signal-to-noise at higher values of 2θ, where high-quality data are critical for accurate crystal structure refinement. This is readily achieved using an open-flow N_2_ gas cooler, mounted coaxially with the capillary (Fig. 3 ▸). When using a well-maintained cooling device, icing-up of the capillary is rarely an issue but can be easily detected by the presence of the three particularly sharp diffraction lines in the range 22–26° 2θ that are characteristic of ice Ih.

Degradation of the sample upon exposure to a laboratory X-ray source is rare. At synchrotron sources, X-ray damage to mol­ecular organic samples is much more likely due to the intense incident beam and strategies to mitigate damage, such as translation of the capillary in the beam (to expose fresh sample for each rapid data collection) and robotic sample changers (to replace fully exposed capillaries with fresh ones), are essential. Concerns regarding sample solvent loss or moisture gain are addressed as outlined in Fig. 1 ▸.

## Data preparation

### Background subtraction

After reading diffraction data into the crystal structure solution program *DASH*, background subtraction is an essential prerequisite to successful Pawley fitting. The program gives a preview of its estimated background prior to subtracting it, with adjustable parameters that allow it to be varied. These ensure that the background is well traced, without cutting into the bases of weaker diffraction features at higher 2θ. As it is the background-subtracted data set that is used in all subsequent *DASH* operations, including the crucial step of intensity extraction by Pawley fitting (Section 3.3), particular attention should be paid to fitting the background as accurately as possible.

### Unit-cell determination

In general, accurate positions of ∼20 well-defined diffraction peaks are required to successfully determine the lattice parameters of the unit cell. Although several indexing programs, including *TOPAS*, may return the correct lattice parameters in the presence of a few spurious peaks (*e.g.* from contaminant phases or noise artifacts), indexing should prioritize those peaks that are clearly defined against the background and that exhibit consistent full-width-at-half-maximum (FWHM) values. The position of each peak is best determined by peak fitting in *DASH*, rather than by manually selecting the peak maximum. For refractory indexing cases, the data collection tem­per­a­ture can help resolve peak overlaps: by acquiring a second dataset at a different tem­per­a­ture, differential thermal expansion may separate previously overlapping reflections, improving input line-position accuracy. Data from samples likely to consist of a mixture of phases should be closely examined for peak width variation that may be indicative of contributions not attributable to the phase of inter­est.

Indexing programs such as *DICVOL* (invoked from within *DASH*) typically generate multiple possibilities, ranked in order of a figure of merit. A visual cross-check of the predicted peak positions of the top candidate unit cell against the actual positions of diffraction peaks should be performed and, for a correct solution, each observed peak should correspond to a predicted reflection. The candidate unit cell should be verified using the estimated mol­ecular volume, *V*_mol_, calculated using Hofmann’s method (Hofmann, 2002 ▸), which has been implemented as a web app (https://hofcalc.streamlit.app/). The ratio *V*_cell_/*V*_mol_ should return a crystallographically-plausible value of *Z*, *e.g.* a ratio of 3.9 strongly suggests *Z* = 4. Following successful indexing, all subsequent analyses should employ the conventional unit-cell setting, to ensure that the final Crystallographic Information File (CIF) meets deposition requirements (Section 7). *PLATON* is strongly recommended for transforming the indexed cell setting to the conventional setting.

### Pawley refinement and intensity extraction

A Pawley refinement in *DASH*, against the fixed-count-time data, should be attempted in a primitive symmorphic space group (*i.e.* a space group without systematic absences) that is consistent with the crystal system, *e.g. P*2 for monoclinic and *P*222 for ortho­rhom­bic. If all the observed diffraction features are well fitted, this is a strong endorsement of the unit cell and crystal system. The *R*_wp_ value obtained should be recorded prior to moving onto space group determination using the probabilistic approach implemented in the *ExtSym* routine within *DASH. ExtSym* analyses the extracted reflection intensities from the Pawley refinement and returns a list of extinction symbols and their associated probabilities (Table 4 ▸). As there may be several space groups that are consistent with one extinction symbol (Looijenga-Vos & Buerger, 2006 ▸), observed space group frequencies for organic com­pounds (Cambridge Crystallographic Data Centre, 2024 ▸) can aid distinction, as can the chiral com­position (*e.g.* enanti­opure *versus* racemic) for com­pounds containing chiral centres.

Once the space group has been determined, a subsequent *DASH* Pawley refinement in this space group should return essentially the same *R*_wp_ value as the initial Pawley refinement performed using the primitive symmorphic space group. In practice, a slight increase in *R*_wp_ is typically seen, due to the reduced number of variable intensities in the second Pawley refinement. It is this second Pawley refinement, in the correct space group, that extracts the correlated integrated intensities that are used by *DASH* for crystal structure solution. It is therefore essential to take particular care with this refinement, to achieve the best possible fit to the data. Note that while *DASH* does not feature the ability to model anisotropic line broadening, this is generally not a significant impediment to structure solution.

Pawley refinement in *TOPAS*, against the high-quality high-resolution VCT dataset, provides final verification of the unit cell and space group. Any unfitted observed diffraction features are indicative of either an incorrect unit cell/space group or the presence of contaminating phase(s) in the sample. It is not uncommon for the crystal structures of the contaminants to be known, *e.g.* residual starting materials from a mechanochemical cocrystal synthesis. In such cases, *TOPAS* can perform a combined refinement, with Rietveld refinement of each contaminant phase alongside Pawley refinement of the unknown cocrystal structure. This yields a phase-pure background-subtracted XY cocrystal dataset, significantly improving the likelihood of successful cocrystal structure solution with *DASH* (see supporting information file SI-1 for an example).

#### Pawley refinement using existing lattice parameters

When the unit cell and space group are known from a prior crystal structure determination at tem­per­a­ture *T*_1_, Pawley refinement of data collected at *T*_2_ can be initiated using the *T*_1_ lattice parameters as starting values. If Δ*T* is substantial, the Pawley refinement may converge to a local minimum if the *T*_2_ lattice parameters differ significantly from their starting *T*_1_ values. To mitigate this risk, the ‘continue_after_convergence’ and ‘val_on_continue’ commands in *TOPAS* enable repeated Pawley refinements from randomized *T*_1_ parameter variations, probing systematically for correct *T*_2_ convergence (see sup­porting information file SI-2 for an example). This systematic parameter exploration can also prove valuable when indexing PXRD data with a dominant zone: allowing a poorly-defined lattice parameter to vary, while fixing those of the dominant zone, often yields correct convergence.

## Crystal structure solution

### 3D model construction

One of the most important aspects of SDPD is the use of prior chemical knowledge, expressed in Z-matrix inter­nal coordinate format,^2^ to com­pensate for the limited amount of structural information in a PXRD pattern. The *DASH* input model contains each fragment in the asymmetric unit (potentially organic neutral mol­ecules; organic cations and anions; inorganic cations and anions) as a Z-matrix. This allows each fragment to be treated as a rigid body (RB) in which the only optimizable variables are the rotatable torsion angles (inter­nal degrees of freedom, DoF) and the position and orientation of the fragments (external DoF). For example, ibuprofen (C_13_H_18_O_2_) has a total of 10 DoF (Z-matrix) com­pared with 99 DoF (Cartesian coordinates). Thus, the Z-matrix ensures efficiency by keeping the number of optimizable variables to a minimum.

*DASH* constructs each Z-matrix from input coordinates and the recommended input coordinate format is MOL2, because it explicitly defines atom types (@<TRIPOS>ATOM), as well as bond types and connectivity (@<TRIPOS>BOND). This ensures that inter­nal DoF are determined correctly in the Z-matrix. With fractional or Cartesian coordinates, the recommendation is always to convert to MOL2 format using, for example, *Mercury*. The input model should include H atoms – they are not used by default in *DASH* structure-factor calculations, but they ease visual inter­pretation of crystal structure solutions and they get used in Z-matrix construction.

Given that all other geometric features remain fixed (covalent bond lengths and angles; non-rotatable torsion angles; aliphatic ring conformations; inter­planar angles; rigid groups), it is critical to start global optimization (GO, Section 4.2) with a geometrically accurate model (the ramifications of inaccuracies for GO are examined in Section 4.5). This is often obtained from a crystal structure or, increasingly, from a gas-phase density functional theory (DFT) geometry optimization of an isolated mol­ecule. It is highly advantageous to know the absolute configuration of each chiral centre and, in the modern era, this is often known from the synthetic pathway.

Regardless of how the input model is generated, it is good practice to check that the covalent bond lengths and angles follow well-established patterns: this can be achieved by evaluating *z*-scores^3^ using the *Mogul* functionality of *Mercury. Mogul* is a knowledge-based library of mol­ecular geometry derived from the Cambridge Structural Database (CSD) and a high *z*-score (≳ 2.0) may indicate a suspect covalent bond length or angle within a model that needs to be addressed.

By default, aliphatic rings are treated as RBs and if the ring conformation is not known *a priori*, then the following two strategies should be considered:

(i) conformer generators [*e.g. GOAT* (de Souza, 2025 ▸), as implemented in *ORCA*, from Version 6] can be used to provide candidate structures for discrete sets of GO runs, *e.g.* four probable aliphatic ring conformations require four corresponding *DASH* input models;

(ii) the alternative approach is to allow optimization of a ring conformation during the GO process by breaking a covalent bond within it, thereby converting it into a series of additional rotatable torsion angles.

The disadvantage of strategy (i) is obvious; *Z*′ > 1 leads to a combinatorial explosion in the number of *DASH* input models. Although strategy (ii) can significantly increase the number of inter­nal DoF, it negates the need to create multiple models *via* conformer generation. Crucially, the crystal structure solution program *GALLOP* allows the length of the broken bond to be used to form a restraint that forces the distance between the two atoms involved to refine to the known bond length during optimization of the torsion angles in the ring system (this facility is not available in *DASH*). This narrows the search space, such that the increased number of DoF is much less of a concern. On balance, the effectiveness of strategy (ii) makes it a very strong recommendation when dealing with aliphatic rings of unknown conformation (Spill­man *et al.*, 2022 ▸).

### Global optimization

*DASH* solves a crystal structure by optimizing the position,^4^ orientation and conformation of each fragment in the asymmetric unit, using the agreement between the observed (as extracted during the Pawley fit) and calculated reflection intensities as a figure of merit (FOM) – the lower the FOM, the better the agreement between the observed and calculated data. Note that DoF are optimized against the PXRD data only – conformational and lattice energies play no part.

*DASH* uses a simulated annealing (SA) algorithm to perform the GO, in which the crystal structure can be visualized as a hypothetical particle moving within a hyperspace dictated by the structure’s *N* variable parameters, *i.e.* the positional, orientational and torsional DoF. For efficiency, it uses a χ^2^ FOM. As no single SA run is guaranteed to find the χ^2^ global minimum on the *N*-dimensional χ^2^ hypersurface, multiple SA runs are needed, each commencing from a different random start point on the hypersurface.

The recommended number of SA runs and SA moves depends on the com­plexity of the problem. With a small total DoF, 20 runs of 10 million moves each is a good starting point. As com­plexity increases and DoF approaches ∼40, 900 runs of 20 million moves each is not unreasonable and it becomes advisable to execute the runs in parallel using *MDASH* (‘*M*’ indicating multicore), *e.g. MDASH* running on an eight-core CPU enables 1000 SA runs to be executed as 125 runs per core.

Use of the Kabova settings (Kabova *et al.*, 2017*a* ▸) for the SA algorithm in *DASH* is strongly recommended, as they have been shown to be significantly more effective than the Version 3.3 defaults. Similarly, for mol­ecules with large numbers of torsion angles (typically > 10), the use of the MDB (Mogul Distribution Bias) settings (Kabova *et al.*, 2017*b* ▸) can improve the chance of obtaining a good solution, by biasing the torsion angle space searched by the SA to areas that are probable, based on *Mogul* database information. It is worth noting that these boundaries apply only to the SA part of a *DASH* run and are lifted for the simplex optimization that occurs at the end of a *DASH* run.

Users with access to com­puters equipped with modern graphics processing units (GPUs) are directed to the *GALLOP* program that operates on the files generated by a *DASH* Pawley refinement and also uses the Z-matrix inter­nal coordinate format. Like *DASH*, *GALLOP* minimizes the χ^2^ FOM, but employs a different approach to optimization, combining a fast local optimizer with a particle swarm optimizer.^5^ The combination of the local optimization steps fol­lowed by one particle swarm step make up a single *GALLOP* iteration. During SDPD, iterations continue until either a target value of χ^2^ is achieved, a set number of iterations has been com­pleted or the user inter­rupts the program. This approach significantly improves both the speed, and frequency of success, of solving com­plex mol­ecular organic crystal structures. At the time of writing, for optimal performance, an NVIDIA GPU-based card with a com­puting capability of ≥ 3.5 (NVIDIA, 2025 ▸) and ≥ 6 GB of on-board memory is recommended.

### Figures of merit

While function minimization in *DASH* is performed using a correlated integrated intensities χ^2^ FOM for speed and efficiency, use of the output profile χ^2^ FOM is more intuitive as it can be directly related to the profile χ^2^ that was obtained at the end of the Pawley refinement process: this latter χ^2^ represents the best χ^2^ that can be achieved by the SA process. Therefore, each time a new intensity χ^2^ minimum is found, the profile χ^2^_SA_ is updated. How close the value of χ^2^_SA_/χ^2^_Pawley_ gets to 1 depends upon both the PXRD data and the model accuracy but, as a general rule, any solution with a χ^2^_SA_/χ^2^_Pawley_ < 5 is worthy of close inspection. Use of the simplex option in *DASH* is also strongly recommended: a simplex optimization that rapidly reduces χ^2^_SA_ to the lowest value in that vicinity of space is invoked either when χ^2^_SA_/χ^2^_Pawley_ falls to less than a user-set value (the default is 5), or when all available SA moves are exhausted.

In general, the values of correlated integrated intensities χ^2^ and profile χ^2^ move in step; the solution with the lowest correlated integrated intensities χ^2^ will have the lowest profile χ^2^. Occasionally, when Pawley refinement has been problematic (*e.g.* with noisy/weak data, or data with significant anisotropic line broadening) and the final Pawley fit is not particularly good, this lock-stepping of χ^2^ values does not hold, and it is better to consider the profile χ^2^ value alone when considering the best solution to examine.

### Troubleshooting

**Figure 4:**
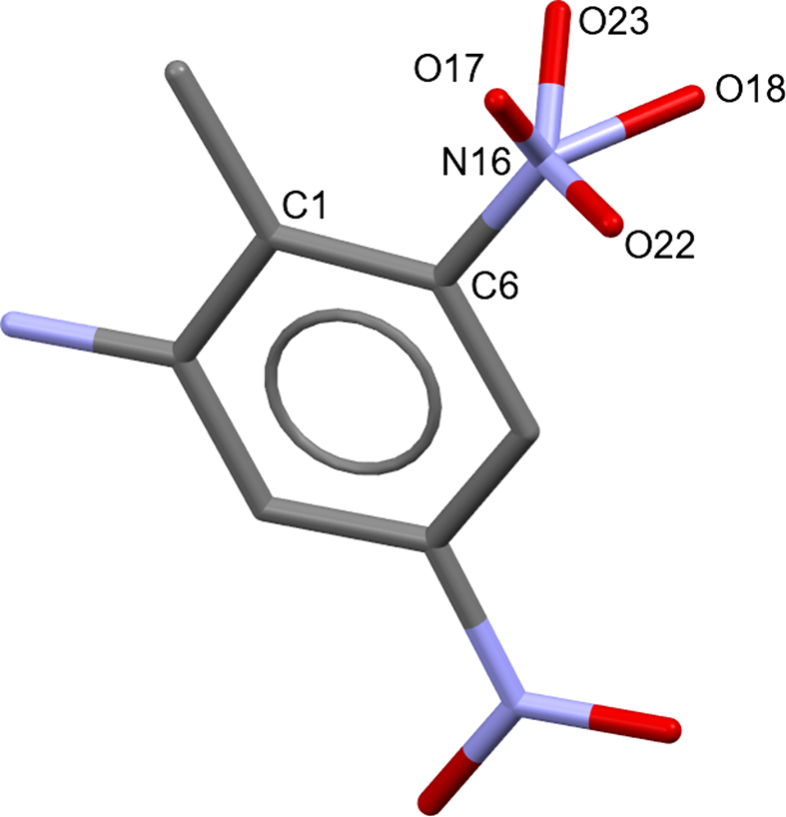
The crystal structure of 2-amino-4,6-di­nitro­toluene was initially determined from PXRD data at 293 K (Graham *et al.*, 2004 ▸). The fit to the data improved significantly upon modelling disorder in the C6-nitro group, but the limited quality and resolution of the PXRD data precluded accurate refinement of the site-occupancy factors (SOFs). To address this, each O atom was modelled over two positions, with SOFs constrained to sum to 1.0 (atom numbers correspond with Table 5 ▸). The structure was solved with fixed SOF values and the solution yielding the best agreement with the data was selected as most probable. Subsequent single-crystal determination at the same tem­per­a­ture (structure shown above) con­firmed the disorder model. In both determinations, each O atom of the 6-nitro group is disordered between a high-occupancy site (O23: 80% single crystal, 70% PXRD) and a low-occupancy site, with excellent agreement in the positions of the high-occupancy sites. The observed structural differences can be attributed to the disparity in available diffraction data – the single-crystal analysis utilized 1111 observed reflections extending to ∼0.8 Å resolution, while the PXRD dataset contained just 146 unique reflections with a maximum resolution of ∼1.8 Å. This represents an early application of *DASH* for crystal structure determination, predating the now-standard incorporation of Rietveld refinement and periodic DFT-D geometry optimization into the workflow.

When GO fails to return plausible structure solutions, consider these strategy modifications:

(i) prepare a different sample and recollect the diffraction data – options include using a new batch of powder (*i.e.* different to that which produced the initial sample) and recollecting at both room tem­per­a­ture and 150 K;

(ii) carry out the Pawley fit again in *DASH*, but to slightly lower resolution;

(iii) check for unfitted observed diffraction features that might indicate either an incorrect unit cell/space group or the presence of contaminating phase(s) in the sample;

(iv) check for issues with the *DASH* input model, *e.g.* test al­ternative conformations of aliphatic rings/pyramidal N atoms and consider the possibility of disorder (Fig. 4 ▸ and Table 5 ▸);

(v) include MDB settings in the *DASH* GO run set up;

(vi) include a March–Dollase correction (Dollase, 1986 ▸) for PO in the *DASH* set-up.

### Candidates for Rietveld refinement

As a general rule, any *DASH* solution with χ^2^_SA_/χ^2^_Pawley_ < 5 merits close inspection. This involves assessing the difference profile and validating the crystal packing, *i.e.* ensuring the absence of steric clashes, the presence of chemically reasonable hydrogen-bond geometry and consistency with com­parable CSD structures. The two key scenarios where χ^2^_SA_/χ^2^_Pawley_ < 5 are:

(i) plausible crystal packing, with a relatively flat difference profile – this suggests that the GO has located the χ^2^ global minimum;

(ii) plausible crystal packing, with significant peaks and troughs in the difference profile – this suggests that the GO has converged to a χ^2^ local minimum, proximate to the global minimum.

Such local minima typically arise from inaccuracies in the GO input model’s fixed geometric parameters, *e.g.* an inter­planar angle that optimized to 0° in the gas phase may adopt a significantly non-zero value in the crystal structure, due to non-covalent inter­actions. While PXRD patterns contain limited structural information, such discrepancies are often evident in high-quality difference profiles. Accordingly, the geometry should be updated by optimizing the output of a preliminary Rietveld refinement using dispersion-corrected periodic DFT (periodic DFT-D), then using this updated geometry to create fresh input models for more GO runs.

If the best χ^2^_SA_/χ^2^_Pawley_ > 5, it should not be rejected without first inspecting the crystal packing and, if it is plausible, then proceeding as described above. Once a promising solution has been identified, structure refinement can begin.

## Crystal structure refinement

When refining a crystal structure using the Rietveld method, the high-quality high-resolution VCT dataset collected from the sample should be used. First, a Pawley refinement against this data should be carried out to obtain the best possible fit, and its *R*_wp(Pawley)_ value noted. Anisotropic peak broadening should be introduced at this stage, if required. Next, a Rietveld refinement of the best candidate crystal structure should be performed, keeping all the variable fit parameters from the Pawley refinement fixed at their refined values, except for those associated with the background. This initial Rietveld fit, in which only the overall scale factor, overall tem­per­a­ture factor *B*_iso_ (a single variable with a starting value of 3 for all non-H atoms, with *B*_iso_ for all H atoms set to be 1.2 times the variable value) and background parameters are refined^6^ should return an *R*_wp_ value close to the *R*_wp_ obtained in the Pawley refinement. Typically, for a candidate structure that is close to the correct crystal structure, the ratio *R*_wp(Rietveld)_/*R*_wp(Pawley)_ will be less than 3. At this stage, there are a number of possible approaches to refining the crystal structure: for these approaches outlined in Sections 5.1–5.3, it is assumed that the model being refined is a com­plete description of the asymmetric unit.

### Free-atom refinement

For anything other than a structure com­prised of only a few atoms and very high-quality atomic resolution data, Rietveld free refinement of atomic coordinates, even when restricted to non-H atoms with H atoms treated as riding, is not recommended. Due to the typical dearth of intensity values, the limited spatial resolution and the inevitable experimental errors in the measured data, the least-squares refinement minimizes the *R*_wp_ value at the expense of chemical sense, resulting in a crystal structure with unrealistic mol­ecular geometry.

### Restrained refinement

In a Rietveld restrained refinement, minimization of *R*_wp_ is carried out subject to a series of bond distance, bond angle, torsion angle and other restraints, *e.g.* aromatic ring flattening. These restraints are derived from the starting model and can be weighted, individually and overall, against the diffraction data. Deciding on the correct weighting of restraints against data can be challenging; at one extreme, the problem becomes akin to a free-atom Rietveld refinement, while at the other, it is akin to an RB Rietveld refinement, though without the benefits of an RB definition.

After every cycle of refinement, the structure needs to be carefully examined to see if the weighting scheme needs updating in order to achieve a good balance between im­prov­ing the fit to the data while maintaining chemical sense. While this approach is perfectly effective, the use of RBs (Section 5.3) ultimately provides a more easily controlled route to a satisfactory final structure.

### Rigid-body refinement

The GO-based structure solving procedure in *DASH* utilizes an RB approach, starting from an accurate input model. It therefore makes sense to carry over that same RB definition into the Rietveld refinement and use it as the basis for refinement against the high-quality high-resolution VCT dataset. The solved structure is therefore refined in a least-squares fit in which the variables are now the overall scale factor, background, overall *B*_iso_, the position and orientation of each RB, plus the same torsion angle values that were optimized in the *DASH* runs, ultimately bringing it to the closest minimum in *R*_wp_ space. The key geometric features of the mol­ecule, which were validated at the outset of the structure solving process, are therefore preserved. Unsurprisingly, with a relatively small number of refinable parameters, the *R*_wp_ value does not usually drop much from the ‘scale-only’ *R*_wp_ value. Note that, if the input model is inaccurate with respect to any of the fixed geometric parameters, this inaccuracy will not be resolved in an RB Rietveld refinement where the only inter­nal DoF are the original variable torsion angle values. It is straightforward to flag additional variables, such as a particular bond angle, within the RB description and repeat the refinement, but such small discrepancies are generally better addressed in the subsequent structure verification steps (Section 6). It is also worth checking to see if the addition of a PO correction (either March–Dollase or spherical harmonics^7^) to the refinement brings about a significant improvement in the *R*_wp_ value and an improvement in the quality of the refined structure.

#### Rigid bodies in *TOPAS*

*TOPAS* is now one of the most widely-used programs for Rietveld refinement, but implementing an RB refinement can be daunting, as it involves several steps to set up. The structure being refined initially consists of a set of fractional atomic coordinates {**r**_1_, **r**_2_, …, **r**_*n*_}, where **r**_*i*_ = (*x*_*i*_, *y*_*i*_, *z*_*i*_), whereas the RB description of the mol­ecule consists of a set of *n* atoms whose relative positions are described by a set of covalent bond lengths, angles and torsion angles. The RB description first has to be mapped onto the fractional coordinates of the *DASH* crystal structure solution before the refinement can proceed based on refining the inter­nal and external DoF of the RB. This mapping is done by creating a new set of fractional atomic coordinates {**r′**_1_, **r′**_2_, …, **r′**_*n*_} that are tied to the RB description *via* a unique set of atom names, and instructing *TOPAS* to perform an ‘only_penalties’-type refinement that minimizes the distances between corresponding atoms in the two sets of coordinates. At the end of this minimization, the RB is superimposed upon the original coordinates, which can then be deleted and refinement of the structure continued using the RB description; the associated set of fractional coordinates {**r′**_1_, **r′**_2_, …, **r′**_*n*_} is updated at the end of each cycle. Typically, only the position and orientation of the RB, plus the variable torsion angles, are refined from this point onwards. For users of *DASH*, this set-up process is made considerably easier by use of a web app (https://zm-to-inp.streamlit.app/) that converts a *DASH* Z-matrix file into a corresponding RB definition in *TOPAS* INP format: the resultant INP file contains clear instructions on how to map the RB definition onto the actual coordinates. Note that the ‘zm-to-inp’ process must be run on a Z-matrix generated from the solved *DASH* structure, to ensure that it encodes the correct mol­ecular conformation of that structure.

## Crystal structure verification

Traditionally, *checkCIF* marked the final pre-publication step for crystal structures, with significant issues in the Rietveld CIF typically resolved through further refinement. Now, a more robust approach has emerged – periodic DFT-D geometry optimization, bridging the gap between RB Rietveld refinement and *checkCIF* validation. The structure optimizes to the nearest energy minimum in a calculation that treats the crystal structure in its entirety and is independent of the diffraction data.

A periodic DFT-D calculation is typically executed in two stages (van de Streek & Neumann, 2010 ▸). The RB Rietveld-refined crystal structure is first geometry-optimized, varying atomic coordinates within fixed lattice parameters, then the optimized structure is further optimized by varying both atomic coordinates and lattice parameters, while ensuring that the Bravais lattice is not allowed to change. The resultant change in lattice parameters means that the output atomic coordinates cannot be recycled directly into another RB Rietveld refinement. However, the geometry of the optimized asymmetric unit can be used as the basis of a final RB Rietveld that takes advantage of the geometry improvements provided by periodic DFT-D.

The key verification of the crystal structure’s correctness involves overlaying the final RB Rietveld-refined structure on its fully DFT-D-optimized equivalent and calculating a 15-mol­ecule Cartesian root-mean-square deviation value (RMSD15) for the overlay, excluding H atoms. An RMSD15 of less than 0.35 Å (a well-established threshold for SDPD) is indicative of minimal atomic displacement and an accurate crystal structure (van de Streek & Neumann, 2014 ▸).

**Figure 5:**
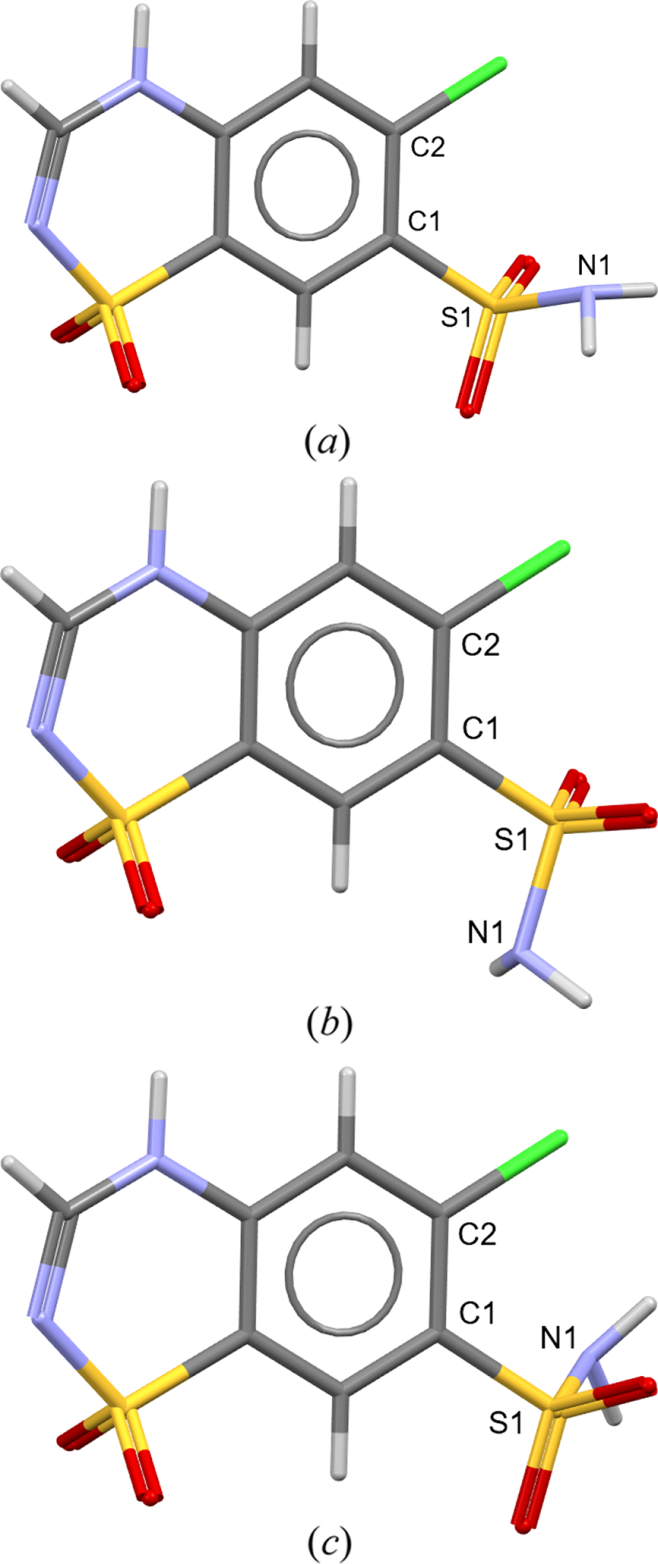
In a *DASH* crystal structure solution of chloro­thia­zide, the C2—C1—S1−N1 torsion angle may adopt values of *x*° (*a*), *x* + 120° (*b*) or *x* − 120° (*c*), due to the negligible difference in X-ray scattering between =O (8 e^−^) and –NH_2_ (9 e^−^). Consequently, the presence of all three –S(=O)_2_–NH_2_ orientations in the ensemble of *DASH* output solutions is to be expected. This type of ambiguity also arises with the rotation of a carboxyl group (O=C—OH, typically two orientations at *x*° and *x* + 180°) and the orientation of a mesylate anion [CH_3_—S(=O)_2_—O^−^, up to four orientations]. While Rietveld refinement against a high-quality high-resolution VCT dataset may be able to resolve these cases, hydrogen-bond geometry analysis and periodic DFT-D calculations offer superior discrimination.

The capacity of periodic DFT-D to address key limitations of a purely diffraction-based approach (such as resolving H-atom positional ambiguities and issues of the type shown in Fig. 5 ▸) has established it as the benchmark method for verifying SDPD crystal structures. For com­prehensive guidance on applying periodic DFT-D to mol­ecular organic crystal structures (particularly in an SDPD context), the work of van de Streek & Neumann (2010 ▸, 2014 ▸) is strongly recommended.

At present, periodic DFT-D remains com­putationally intensive, typically requiring high-performance com­puting resources for routine execution. Software packages such as *Quantum ESPRESSO*, optimized specifically for such environments, represent the current state-of-the-art for these calculations and while not essential for crystal structure verification, the use of periodic DFT-D is very highly recommended. Table 6 ▸ lists some key input file parameters for *Quantum ESPRESSO* that the authors have found to be effective in verifying SDPD crystal structures.

## Crystal structure validation

A CIF can be checked online (https://checkcif.iucr.org/) or locally, using *PLATON* for a detailed chemical and crystallographic analysis of the structure and *enCIFer* to identify and correct syntax/format violations (Allen *et al.*, 2004 ▸). Crystal structures derived from PXRD data typically trigger multiple *checkCIF* alerts, most of which are readily addressed, *e.g.* the *TOPAS* Pawley refinement provides estimated standard deviations (e.s.d.’s) for lattice parameters. However, warnings about missing error estimates on atomic coordinates require special consideration. In RB Rietveld refinement, the least-squares optimization directly refines inter­nal and external DoF, yielding e.s.d.’s for these parameters, not for the atomic coordinates. To resolve this, a final cycle of RB Rietveld refinement in *TOPAS*, with bootstrap error analysis enabled *via* the ‘bootstrap_errors’ directive, generates a CIF that includes e.s.d.’s on the atomic coordinates.

## Conclusion and perspectives

Microfocus X-ray sources have dramatically reduced the crystal size requirements for single-crystal diffraction and the advent of commercial laboratory electron diffractometers has reduced those requirements further still. That said, laboratory PXRD remains an easily-accessible and well-defined route to both bulk sample characterization and, in combination with periodic DFT-D, accurate SDPD.

## Supplementary Material

SI-1 ZIP file with TOPAS pattern extraction demo. DOI: 10.1107/S2053229625008046/ky3231sup1.zip

SI-2: Word file with text on use of TOPAS 'val_on_continue'. DOI: 10.1107/S2053229625008046/ky3231sup2.docx

## Figures and Tables

**Table 1:** Summary of software used in this work

Software	Application	Reference
*DASH*	Indexing*^*a*^*, space-group determination*^*b*^*	David *et al.* (2006 ▸)
	Pawley refinement, crystal structure solution	
*MDASH^*c*^*	Crystal structure solution	Griffin *et al.* (2009 ▸)
*GALLOP*	Crystal structure solution	Spillman & Shankland (2021 ▸)
*TOPAS-Academic^*d*^*	Indexing, Pawley refinement, Rietveld refinement	Coelho (2018 ▸)
*MarvinSketch*	3D model construction	ChemAxon (2025 ▸)
*ORCA*	Mol­ecular geometry optimization	Neese (2025 ▸)
CSD*^*e*^*	Primary CIF source	Groom *et al.* (2016 ▸)
*ConQuest*	CSD 3D searching	Bruno *et al.* (2002 ▸)
*Mercury*	Structure visualization	Macrae *et al.* (2020 ▸)
*Mogul*	Mol­ecular geometry validation	Bruno *et al.* (2004 ▸)
*Quantum ESPRESSO*	Crystal structure geometry optimization	Carnimeo *et al.* (2023 ▸)
*PLATON*	Crystal structure validation	Spek (2020 ▸)

Notes: (*a*) *Via DASH* inter­face to *DICVOL91* (Boultif & Louër, 1991 ▸). (*b*) *Via DASH* implementation of *ExtSym* (Markvardsen *et al.*, 2008 ▸). (*c*) Enables *DASH* to utilize multiple CPU cores. (*d*) Refinements reported here used *TOPAS-Academic*, but the same methodologies apply to the commercially licensed version, *TOPAS* (Bruker AXS). (*e*) Cambridge Structural Database.

**Table 2:** Two recommended data collection schemes for laboratory PXRD data

Time (hr)	Count type	Step (°)	Range (°)	Resolution (Å)	Purpose
2	Fixed	0.017	2.5–40	2.25	Indexing, Pawley refinement, space group determination, global optimization
12	Variable	0.017	2.5–70	1.35	Pawley and Rietveld refinement

**Table 3:** A VCT data collection scheme for Rietveld-quality data

Start (°)	End (°)	Step (°)	Count time per step (s)
2.5	22	0.017	2
22	40	0.017	4
40	55	0.017	15
55	70	0.017	24

**Table 4:** Sample output from *ExtSym*, using Pawley-extracted intensity data fitted in *P*222 (space group number in parentheses) Space group *P*2_1_2_1_2_1_ is the most probable. Note for extinction symbol *P*–*n*–, there are two possible matching space groups, one centrosymmetric and one non-centrosymmetric.

Extinction symbol	Log-probability score	Possible space groups
*P*2_1_2_1_2_1_	20.9031	*P*2_1_2_1_2_1_ (19)
*P*–*n*–	17.4761	*Pmn*2_1_ (31), *Pmnm* (59)
*P*2_1_2_1_–	14.524	*P*2_1_2_1_2 (18)
*P*2_1_–2_1_	11.3921	*P*2_1_22_1_ (18)
*P*–2_1_2_1_	9.03614	*P*22_1_2_1_ (18)
*P*2_1_––	8.43998	*P*2_1_22 (17)
*P*–2_1_–	6.08402	*P*22_1_2 (17)
*P*––2_1_	2.95213	*P*222_1_ (17)

**Table 5:** Z-matrix used in the crystal structure determination of 2-amino-4,6-di­nitro­toluene (Fig. 4 ▸) This empirical geometry predates the now-standard use of gas-phase DFT geometry optimization to produce a *DASH* input model, but illustrates the key parameters: (*a*) inter­nal mol­ecular geometry, *e.g.* C4—C3 = 1.40 Å, C4—C3—C2 = 120.0° and C4—C3—C2—C1 = 0.0°; (*b*) optimization flags, 0 = fixed and 1 = variable, in columns 3, 5 and 7; (*c*) site-occupancy factor, in column 12.

Atom	Length	Ref.	Angle	Ref.	Torsion	Ref.	Bond to	Angle to	Torsion to	*B*_iso_	SOF
C1	0.00	0	0.0	0	0.0	0	–	–	–	3	1.0
C2	1.40	0	0.0	0	0.0	0	1	–	–	3	1.0
C3	1.40	0	120.0	0	0.0	0	2	1	–	3	1.0
C4	1.40	0	120.0	0	0.0	0	3	2	1	3	1.0
C5	1.40	0	120.0	0	0.0	0	4	3	2	3	1.0
C6	1.40	0	120.0	0	0.0	0	5	4	3	3	1.0
C7	1.50	0	120.0	0	180.0	0	1	2	3	3	1.0
H8	1.00	0	109.5	0	90.0	0	7	1	2	3	1.0
H9	1.00	0	109.5	0	210.0	0	7	1	2	3	1.0
H10	1.00	0	109.5	0	330.0	0	7	1	2	3	1.0
H11	1.00	0	120.0	0	180.0	0	3	2	1	3	1.0
H12	1.00	0	120.0	0	180.0	0	5	6	1	3	1.0
N13	1.33	0	120.0	0	180.0	0	2	3	4	3	1.0
H14	1.00	0	120.0	0	0.0	0	13	2	3	3	1.0
H15	1.00	0	120.0	0	180.0	0	13	2	3	3	1.0
N16	1.46	0	120.0	0	180.0	0	6	5	4	3	1.0
O17	1.23	0	120.0	0	0.0	1	16	6	1	3	0.5
O18	1.23	0	120.0	0	180.0	0	16	6	17	3	0.5
N19	1.46	0	120.0	0	180.0	0	4	5	6	3	1.0
O20	1.23	0	120.0	0	0.0	1	19	4	5	3	1.0
O21	1.23	0	120.0	0	180.0	0	19	4	20	3	1.0
O22	1.23	0	120.0	0	90.0	1	16	6	1	3	0.5
O23	1.23	0	120.0	0	180.0	0	16	6	22	3	0.5

**Table 6:** Some recommended parameters for the geometry optimization of mol­ecular organic crystal structures using periodic DFT-D as implemented in *Quantum ESPRESSO*

Item	Value
Functional	Perdew–Burke–Ernzerhof
Pseudopotentials	Projector augmented-wave
Dispersion correction	Grimme DFT-D3
k-point sampling	Automatic
Kinetic energy cutoffs for plane waves	50 Ry
Kinetic energy cutoffs for charge density	400 Ry
Convergence threshold for total energy	0.0001 au
Convergence threshold for charge density	0.001 au

## Footnotes

1 Flat-plate reflection geometry is not recommended for mol­ecular organic SDPD, due to the higher likelihood of deleterious PO and sample transparency effects.

2 The Z-matrix originated in quantum chemistry for defining mol­ecular geometries using inter­nal coordinates (covalent bond lengths, angles and torsion angles), rather than Cartesian coordinates.

3 *z* = |*x* – μ|/σ, where *x* is an observed value, μ is the population mean and σ is the population standard deviation.

4 The number of DoF is reduced if crystallographic special positions are being used and these are relatively straightforward to implement in structure solution and refinement.

5 A particle is an individual crystal structure and a swarm is a set of trial crystal structures.

6 For simplicity, this is referred to as a ‘scale-only’ refinement.

7 High-order spherical harmonics corrections can artificially com­pensate for other model deficiencies, lowering *R*_wp_ without improving the accuracy of the crystal structure. Accordingly, it is strongly recommended that any such corrections are applied judiciously.

## References

[bb1] Allen, F. H., Johnson, O., Shields, G. P., Smith, B. R. & Towler, M. (2004). *J. Appl. Cryst.* **37**, 335–338.

[bb2] Boultif, A. & Louër, D. (1991). *J. Appl. Cryst.* **24**, 987–993.

[bb3] Bruno, I. J., Cole, J. C., Edgington, P. R., Kessler, M., Macrae, C. F., McCabe, P., Pearson, J. & Taylor, R. (2002). *Acta Cryst.* B**58**, 389–397. 10.1107/s0108768102003324 12037360

[bb4] Bruno, I. J., Cole, J. C., Kessler, M., Luo, J., Motherwell, W. D. S., Purkis, L. H., Smith, B. R., Taylor, R., Cooper, R. I., Harris, S. E. & Orpen, A. G. (2004). *J. Chem. Inf. Comput. Sci.* **44**, 2133–2144. 10.1021/ci049780b 15554684

[bb5] Cambridge Crystallographic Data Centre (2024). https://www.ccdc.cam.ac.uk/media/CSD-Space-Group-Statistics-Space-Group-Frequency-Ordering-2024.pdf.

[bb6] Carnimeo, I., Affinito, F., Baroni, S., Baseggio, O., Bellentani, L., Bertossa, R., Delugas, P. D., Ruffino, F. F., Orlandini, S., Spiga, F. & Giannozzi, P. (2023). *J. Chem. Theory Comput.* **19**, 6992–7006. 10.1021/acs.jctc.3c00249 PMC10601483 37523670

[bb7] ChemAxon (2025). *Marvin*. https://www.chemaxon.com/.

[bb8] Coelho, A. A. (2018). *J. Appl. Cryst.* **51**, 210–218.

[bb9] David, W. I. F., Shankland, K., van de Streek, J., Pidcock, E., Motherwell, W. D. S. & Cole, J. C. (2006). *J. Appl. Cryst.* **39**, 910–915.

[bb10] de Souza, B. (2025). *Angew. Chem. Int. Ed.* **64**, e202500393.

[bb11] Dollase, W. A. (1986). *J. Appl. Cryst.* **19**, 267–272.

[bb12] Graham, D., Kennedy, A. R., McHugh, C. J., Smith, W. E., David, W. I. F., Shankland, K. & Shankland, N. (2004). *New J. Chem.* **28**, 161–165.

[bb13] Griffin, T. A. N., Shankland, K., van de Streek, J. & Cole, J. (2009). *J. Appl. Cryst.* **42**, 360–361.

[bb14] Groom, C. R., Bruno, I. J., Lightfoot, M. P. & Ward, S. C. (2016). *Acta Cryst.* B**72**, 171–179. 10.1107/S2052520616003954 PMC4822653 27048719

[bb15] Hofmann, D. W. M. (2002). *Acta Cryst.* B**58**, 489–493. 10.1107/s0108768101021814 12037338

[bb16] Hull, A. W. (1917). *Phys. Rev.* **10**, 661–696.

[bb17] Kabova, E. A., Cole, J. C., Korb, O., López-Ibáñez, M., Williams, A. C. & Shankland, K. (2017*a*). *J. Appl. Cryst.* **50**, 1411–1420.

[bb18] Kabova, E. A., Cole, J. C., Korb, O., Williams, A. C. & Shankland, K. (2017*b*). *J. Appl. Cryst.* **50**, 1421–1427.

[bb19] Le Bail, A., Duroy, H. & Fourquet, J. L. (1988). *Mater. Res. Bull.* **23**, 447–452.

[bb20] Looijenga-Vos, A. & Buerger, M. J. (2006). In *International Tables for Crystallography*, Vol. A, *Space-group symmetry*, edited by Th. Hahn. Chester: International Union of Crystallography.

[bb21] Macrae, C. F., Sovago, I., Cottrell, S. J., Galek, P. T. A., McCabe, P., Pidcock, E., Platings, M., Shields, G. P., Stevens, J. S., Towler, M. & Wood, P. A. (2020). *J. Appl. Cryst.* **53**, 226–235. 10.1107/S1600576719014092 PMC6998782 32047413

[bb22] Markvardsen, A. J., Shankland, K., David, W. I. F., Johnston, J. C., Ibberson, R. M., Tucker, M., Nowell, H. & Griffin, T. (2008). *J. Appl. Cryst.* **41**, 1177–1181.

[bb23] Neese, F. (2025). *Wiley Interdiscip. Rev.: Comput. Mol. Sci.* **15**, e70019.

[bb24] NVIDIA (2025). https://developer.nvidia.com/cuda-gpus.

[bb25] Pawley, G. S. (1981). *J. Appl. Cryst.* **14**, 357–361.

[bb26] Rietveld, H. M. (1969). *J. Appl. Cryst.* **2**, 65–71.

[bb27] Shankland, K. (2019). In *International Tables of Crystallography*, Vol. H, *Powder diffraction*, edited by C. J, Gilmore, J. A. Kaduk & H. Schenk. Chester: International Union of Crystallography.

[bb28] Spek, A. L. (2020). *Acta Cryst.* E**76**, 1–11. 10.1107/S2056989019016244 PMC6944088 31921444

[bb29] Spillman, M. J. & Shankland, K. (2021). *CrystEngComm* **23**, 6481–6485.

[bb30] Spillman, M. J., Shankland, N. & Shankland, K. (2022). *Cryst­EngComm* **24**, 4551–4555.

[bb31] Streek, J. van de & Neumann, M. A. (2010). *Acta Cryst.* B**66**, 544–558. 10.1107/S0108768110031873 PMC2940256 20841921

[bb32] Streek, J. van de & Neumann, M. A. (2014). *Acta Cryst.* B**70**, 1020–1032. 10.1107/S2052520614022902 PMC4468513 25449625

