# Supplementary material for: A good-practice guide to solving and refining mol­ecular organic crystal structures from laboratory powder X-ray diffraction data
Source: Acta Crystallogr C Struct Chem. 2025 Sep 17;81(Pt 10):559–69. doi: 10.1107/S2053229625008046 (PMC12497095; doi:10.1107/S2053229625008046)
Supplement: Supplementary file 1 [file c-81-00559-sup1.zip › Readme.docx]

**SI-1 For Shankland et al, “A good practice guide…”**

**Extracting single component data from a phase mixture data using *TOPAS***

This ZIP file contains files with an example of how to extract a “phase pure” pattern from PXRD data collected from a phase mixture, using a combination of Pawley + Rietveld in *TOPAS*.  In the example, there is a new phase (that of a co-xtal generated by mechanochemical means) and contributions from its two known starting phases; we wish to generate PXRD data that corresponds to the contribution from the new phase only.

The general approach is to:

- Fit the collected diffraction data as well as possible by carrying out a simultaneous two-phase Rietveld / single-phase Pawley fit
- Copy the *TOPAS* .out file from the end of this fit to create a new .inp file
- Modify this .inp file as described below and then run a single cycle of least-squares to generate an XY datafile containing only the contribution of the new phase.

Files contained in this ZIP, in addition to this Readme, are:

**SampleE_VCT.xye**: Laboratory PXRD data collected from a 1:1 carbamazepine:indomethacin co-crystal. The data include contributions from the co-crystal and the two starting materials.

**PawleyPlusTwoRiet.inp**: A *TOPAS* input file that when run, fits the PXRD data. This generates an output file called ‘PawleyPlusTwoRiet.out’.

**ExtractSingleCoXtalPhase.inp**: This is the modified version of ‘PawleyPlusTwoRiet.out’ that when run, will generate an XY file with contributions from the co-crystal phase only. Modifications made to the ‘PawleyPlusTwoRiet.out’ file to create the ExtractSingleCoXtalPhase.inp file are as follows:

- Comment out both the contribution from the background and the contributions from the two known phases
- Add ‘iters 0’ immediately after ‘no_LIMIT_warnings’
- Add ‘Out_X_Ycalc("CocrystalPhase.xy")’ immediately after the reflection listing for the co-crystal phase.

**CocrystalPhase.xy**: This is the phase-pure data, generated from the example files, that has been provided for reference purposes. [Using TOPAS-Academic-64 V7.19]

**Summary when dealing with such a scenario**

First fit the PXRD data as best you can, using a combined Pawley/Rietveld approach.

Then take the best OUT file obtained from that fit, comment-out contributions of the background and the known phases, insert the Out_X_Ycalc line, and then do a SINGLE cycle of least squares.

**Runing the example files**

**Stage 1: The combined two-phase Rietveld and single-phase Pawley fit**

Run the ‘PawleyPlusTwoRiet.inp’ *TOPAS* input file (i.e. launch, Set INP file, run) to obtain the overall best fit to the data by simultaneously Rietveld-fitting the two known phases and Pawley-fitting the new phase. With this example set of files, there is no need to then modify the OUT file, as the ‘ExtractSingleCoXtalPhase.inp’ file has already been provided for simplicity.

**Stage 2: Extracting the phase-pure PXRD data**

Run the ExtractSingleCoXtalPhase.inp file (i.e. launch, Set INP file, run) by pressing the
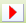
 button.  This will output the calculated contribution of the co-crystal phase, as estimated by the Pawley, to a new file called ‘CocrystalPhase.xy’.
