# Supplementary material for: A good-practice guide to solving and refining mol­ecular organic crystal structures from laboratory powder X-ray diffraction data
Source: Acta Crystallogr C Struct Chem. 2025 Sep 17;81(Pt 10):559–69. doi: 10.1107/S2053229625008046 (PMC12497095; doi:10.1107/S2053229625008046)
Supplement: Supplementary file 2 [file c-81-00559-sup2.docx]

**SI-2 For Shankland et al, “A good practice guide…”**

**Using val_on_continue during Pawley fitting in *TOPAS***

In situations such as the ones described in section 3.3.1, where a Pawley fit is converging to a local minimum, the following approach may prove useful.

In the *TOPAS* input file for the Pawley fit, add

continue_after_convergence

and then after the lattice parameter that you want to adjust, add the command

val_on_continue = Rand (*lower_limit*, *upper_limit*);

before running the Pawley fit.

For example, if it is suspected that the value of the *b* lattice parameter is not converging to the correct value, but that the other lattice parameters are likely accurate, then the following

a 32.964740

b @ 4.901969 val_on_continue = Rand (4.5, 5.5);

c 15.712586

al 90

be 103.20757

ga 90

continue_after_convergence

ensures that when the input file is run, the Pawley executes as normal but on convergence, it assigns a new value in the range 4.5-5.5 Å to the *b* lattice parameter, then re-runs the Pawley. It continues to do so until the run is stopped, at which point the *TOPAS* output file will contain the refined *b* lattice parameter corresponding to the lowest *R*_wp_ obtained during the trials. Other lattice parameters may be ‘nudged’ by the inclusion of further val_on_continue lines.

Each run will execute faster the fewer variables that are included in the Pawley refinement e.g. the background parameters, if they are already well fitted, could be held fixed, as could the peak shape parameters. Finally, including

chi2_convergence_criteria 0.01

will ensure that each Pawley does not spend time drilling right down into every local minimum that it will encounter.
